# Longer or shorter spines: Reciprocal trait evolution in stickleback via triallelic regulatory changes in *Stanniocalcin2a*

**DOI:** 10.1073/pnas.2100694118

**Published:** 2021-07-28

**Authors:** Garrett A. Roberts Kingman, David Lee, Felicity C. Jones, Danielle Desmet, Michael A. Bell, David M. Kingsley

**Affiliations:** ^a^Department of Developmental Biology, Stanford University School of Medicine, Stanford, CA 94305;; ^b^Stanford University School of Humanities and Sciences, Stanford University, Stanford, CA 94305;; ^c^University of California Museum of Paleontology, University of California, Berkeley, CA 94720;; ^d^HHMI, Stanford University School of Medicine, Stanford, CA 94305

**Keywords:** regulatory evolution, stickleback spine length, stanniocalcin, multiallelic polymorphism, supergene

## Abstract

Vertebrates have repeatedly modified skeletal structures to adapt to their environments. The threespine stickleback is an excellent system for studying skeletal modifications, as different wild populations have either increased or decreased the lengths of their prominent dorsal and pelvic spines in different freshwater environments. Here we identify a regulatory locus that has a major morphological effect on the length of stickleback dorsal and pelvic spines, which we term *Maser* (major spine enhancer). *Maser* maps in a closely linked supergene complex that controls multiple armor, feeding, and behavioral traits on chromosome IV. Natural alleles in *Maser* are differentiated between marine and freshwater sticklebacks; however, alleles found among freshwater populations are also differentiated, with distinct alleles found in short- and long-spined freshwater populations. The distinct freshwater alleles either increase or decrease expression of the bone growth inhibitor gene *Stanniocalcin2a* in developing spines, providing a simple genetic mechanism for either increasing or decreasing spine lengths in natural populations. Genomic surveys suggest many recurrently differentiated loci in sticklebacks are similarly specialized into three or more distinct alleles, providing multiple ancient standing variants in particular genes that may contribute to a range of phenotypes in different environments.

## Significance

Across a broad range of species, evolution has modified a common vertebrate body plan to produce endless forms most beautiful. A key unanswered question is whether diverse morphological changes in a common structure arise from modifying different genes or the same genes in different ways. Many natural populations of threespine stickleback have evolved either longer or shorter dorsal and pelvic spines. Here, we identify reciprocal regulatory changes in an ancient enhancer of the bone growth inhibitor, *Stanniocalcin2a*, as an underlying genetic cause. Many other stickleback loci similarly show three or more major classes of variants across populations; we suggest that diverse alleles at key loci may represent a common mechanism for producing diverse phenotypes from a smaller toolkit of genes.

Similar ecological conditions often result in parallel evolution of the same phenotypic traits in independent populations (1–3). However, ecological conditions typically vary in detail between locations, leading to the evolution of interesting phenotypic differences among evolving populations (4, 5). The contrast between convergence and divergence during adaptive radiations has contributed to decades of work seeking to better understand the principles underlying evolution (6, 7).

The threespine stickleback (*Gasterosteus aculeatus*) provides an opportunity to study the mechanisms that contribute to both parallel and divergent evolution. Migratory marine stickleback have been colonizing and adapting to new freshwater environments for millions of years, with the most recent wide-scale radiation occurring in the countless new freshwater environments generated by glacial recession since the last Ice Age, ∼12 Kya (8). Newly derived freshwater populations typically evolve similar phenotypic changes, including reduced bony armor plates and less robust spines. However, characteristic differences also evolve repeatedly among populations in diverse freshwater environments. Decades of work have analyzed the diverging ecological pressures between lakes and streams (9), large and small lakes (10), benthic and limnetic trophic niches within a lake (11), habitats with different water chemistry and light environments (12), and presence or absence of different types of predators (13). Consequently, freshwater stickleback exhibit exceptional phenotypic diversity, including changes in body size, body shape, color, feeding structures, armor plates, and bony dorsal and pelvic spines (8). Despite recent progress on the genetics of some stickleback traits, the molecular mechanisms underlying many phenotypic specializations remain poorly understood.

A key unanswered question is whether diverse evolutionary outcomes occur by modifying different genes in different environments or by modifying the same genes in different ways. Ancient alleles have been identified at particular loci that allow rapid evolution of common marine-freshwater differences by repeated selection of standing variants that already preexist at low frequencies in marine ancestors (14–18). Repeated fixation of preexisting variants favors the reuse of not only the same gene, but also the same freshwater haplotypes in derived populations that share traits. However, it is still not clear whether the distinct phenotypes seen among many freshwater populations are controlled by additional alleles of the same loci that control common marine–freshwater differences, by changes in additional loci, or both.

Phenotypic variability among different stickleback populations is particularly pronounced in the dorsal and pelvic spines for which the species is named. Ossified spines are a key evolutionary innovation that spurred a massive radiation of acanthomorph fish, the remarkably diverse fish group that contains about one-third of all living vertebrate species (19, 20). Threespine stickleback typically have three eponymous dorsal spines, but their length can differ greatly among populations and some populations have more than three while others have fewer than three (8, 21). Paired pelvic fins or hindlimbs are found in both fish and tetrapods. In stickleback, the pelvic fin consists of one fin ray and a large, serrated, locking pelvic spine that articulates with an underlying pelvis and can be raised and lowered as a defense against predators (22). The length of the pelvic spine varies dramatically among stickleback populations, and is sometimes lost entirely (8, 21). Although the *Pitx1* locus has been identified as a major locus controlling major reduction and even complete loss of the pelvic apparatus in stickleback (23, 24), the genes controlling quantitative variation in pelvic spine length in pelvic-complete individuals are still largely unknown. Based on the significance and diversity of dorsal and pelvic spine phenotypes in *Gasterosteus* and other fish, we decided to further investigate the genetic mechanisms underlying spine evolution and development.

## Results

### Identification of a Triallelic Locus Associated with Dorsal and Pelvic Spine Length.

**Fig. 1.**
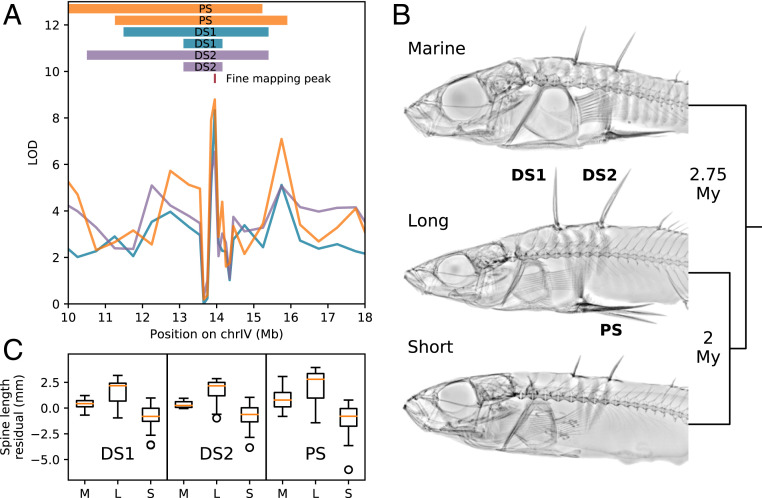
Identification of a triallelic major spine enhancer, *Maser.* (*A*) Genome-wide association mapping of first dorsal spine (DS1, blue trace), second dorsal spine (DS2, purple trace), and pelvic spine (PS, orange trace) length identifies a major spine enhancer, *Maser*, that directly overlaps multiple previous QTL mapping results [visualized here as bars: peak QTL intervals from a Japanese Marine × Paxton Benthic cross (23, 25) and a Little Campbell Marine × Enos Benthic cross (27)]. (*B*) Imputed tree topology of the three *Maser* alleles and approximate divergence times, with X-rays of representative fish from populations with the Marine (M) (Rabbit Slough), Long (L) (Mayer Lake), and Short (S) (Bear Paw Lake) alleles. (*C*) Distributions of DS1, DS2, and PS spine length residuals by genotype at *Maser*.

To identify novel loci controlling adaptations in threespine stickleback, we measured dorsal spine 1 (DS1), dorsal spine 2 (DS2), and pelvic spine (PS) length on 115 fish previously used for whole-genome sequencing (18). These major spines vary significantly in size among stickleback populations, including different freshwater populations (Fig. 1). Comparison of spine lengths and genome sequences across these populations showed that variation in all three spines was strongly associated with a single 18-kb region on chromosome IV (chrIV), 1.1 Mb downstream of the *Eda* gene, which controls armor plate phenotypes (14). The spine length region identified by genome association falls between the two genetically indistinguishable peak markers for a major dorsal spine length quantitative trait locus (QTL) identified by genetic mapping in a previous marine by freshwater cross (25), and also within the peak region controlling pelvic spine length reported in multiple QTL analyses (23, 26, 27) (Fig. 1). We term this locus the *MAjor Spine EnhanceR*, or *Maser*.

Principal component analysis (PCA) of genetic variation at *Maser* identified three distinct clusters: short-spined freshwater (Short), long-spined freshwater (Long), and marine (Marine), with some heterozygous individuals between the clusters (*SI Appendix*, Fig. S2*A*). To more finely assess the genetic differences between these alleles, we PCR-amplified, cloned, and Sanger-sequenced *Maser* from representative Short (Bear Paw Lake, Alaska), Long (Mayer Lake, Haida Gwaii, British Columbia), and Marine (Rabbit Slough, Alaska) populations. We then calculated base substitution rates of 0.0148 between Long and Short, 0.0211 between Short and Marine, and 0.0171 between Long and Marine. This supports a tree topology with both of the freshwater alleles clustered together, suggesting primary divergence of marine and freshwater alleles followed by divergence of the Long and Short freshwater alleles (Fig. 1*B*). A molecular clock calibration derived from divergence between Japanese Marine and Japan Sea stickleback (14, 28) indicates that the initial marine–freshwater divergence at this locus occurred ∼2.75 Mya and that the Long and Short alleles diverged ∼2 Mya. Thus, all three alleles are orders-of-magnitude older than the deglaciation events (∼14 to 5 Kya) that formed the young freshwater habitats in which the Long and Short alleles are currently found (8).

### Fine-Scale Association Mapping of Spine Enhancer.

**Fig. 2.**
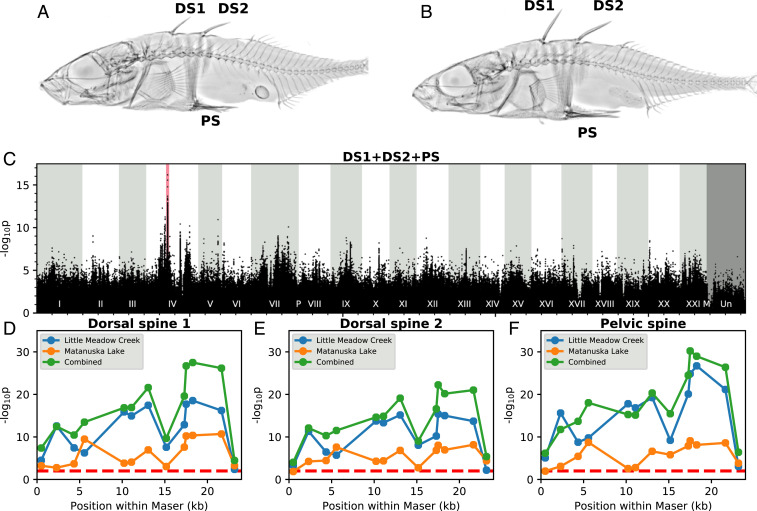
Association mapping of major spine lengths in Little Meadow Creek and Matanuska Lake. (*A* and *B*) X-rays of short-spined (*A*) or long-spined (*B*) fish from Little Meadow Creek. (*C*) Genome-wide association mapping of spine length after whole-genome sequencing of 96 stickleback with extreme spine lengths from Little Meadow Creek and Matanuska Lake. The most strongly associated SNPs are all either in or immediately adjacent to *Maser* (pink). (*D*–*F*) Association between spine length and genotype at 13 PCR-based markers tiled across *Maser*. The dashed red line represents an uncorrected *P* value of 0.01.

To further refine the location of *Maser*, we performed additional whole-genome sequencing on multiple fish from Little Meadow Creek and Matanuska Lake, two Alaskan populations that appeared to contain a mixture of the Long and Short alleles based on the initial sequence survey. Wild fish collected from these locations exhibited a dramatic range of spine lengths (Fig. 2 *A* and *B*). We measured the length of the three dorsal spines, the anal spine, and the right pelvic spine in 335 fish from Matanuska Lake and 359 fish from Little Meadow Creek. We then selected 96 fish with extreme phenotypes to sequence. We generated 12× average coverage for 24 with long spines and 24 with short spines from each population and assessed genotype–phenotype correlation at 2,646,960 variable sites in the stickleback genome with an *F*-test and FastLMM (29). Both methods identified *Maser* as the region most significantly associated with spine length. All SNPs in the genome with *P* values within three orders-of-magnitude of the peak marker are either in or within 10 kb of *Maser* (Fig. 2*C*).

To more sensitively examine the relationship between sequence changes in *Maser* and spine length, we performed fine-scale association mapping on all 694 fish. After regressing on sex and standard body length, the residuals of DS1, DS2, and PS lengths within each fish were highly correlated (*R*^2^ = 0.612, 0.471, 0.500), implying a common regulatory mechanism for the major spines. A 24-kb region around *Maser* (chrIV:13950450 to 13973812 in *gasAcu1-4*) includes 11 large structural variants, 3 TG-repeat arrays, and several hundred SNPs and small indels (*SI Appendix*, Fig. S3). This expands on the original 18-kb window to include a structural variation-rich region with very low mappability for short-read sequencing. We genotyped 13 markers located approximately every 2 kb across this 24-kb region (Dataset S5). In both populations, we observed a highly robust association between the lengths of DS1, DS2, and the PS with marker genotype, with a peak *P* value of 5.82e-34 for combined major spine residuals across all fish (Fig. 2 *D*–*F*). In contrast, we saw weaker association between genotype and length of the much smaller anal spine and DS3, although still statistically significant, suggesting that while the effect of this locus is particularly pronounced on the major spines, it is not limited to them (*SI Appendix*, Fig. S4). At all markers, longer spines were associated with the sequence of the cloned Long allele.

Unlike a traditional F2 cross, association mapping in wild populations can take advantage of potentially hundreds or thousands of generations of recombination, facilitating fine genetic mapping. We detected at least one recombination event in this 24-kb interval in 606 of 694 fish (87.3%). We identified a 5-kb subregion beginning at chrIV:13967840 as being the part of *Maser* most strongly associated with the length of the major spines in both populations. However, the genotype in this 5-kb subregion did not fully explain the effects of *Maser* on spine length; controlling for genotype at the most significant marker, a highly significant association between individual or summed spine length residuals and genotype persisted at other markers within this region (*SI Appendix*, Figs. S4 *C*–*F* and S5 *C* and *F*). Variations at multiple sites within *Maser* probably contribute to its effects on spine length, which is consistent with a growing body of work finding that evolved regulatory differences often reflect separate but tightly linked mutations (30–34).

Notably, genotypes at *Maser* account for a substantial fraction of overall spine length variance in Little Meadow Creek and Matanuska Lake: 32.1% and 20.1%, respectively (Dataset S6). The major phenotypic effect of the *Maser* region is thus comparable in size to the percent variance explained (PVE) by the peak chrIV locus in previous Marine × Short QTL mapping crosses of 25.5 PVE, 28.4 PVE, and 30.2 PVE for PS, DS1, and DS2, respectively (25, 27).

### Tissue-Specific Regulatory Changes in *Stc2a*.

**Fig. 3.**
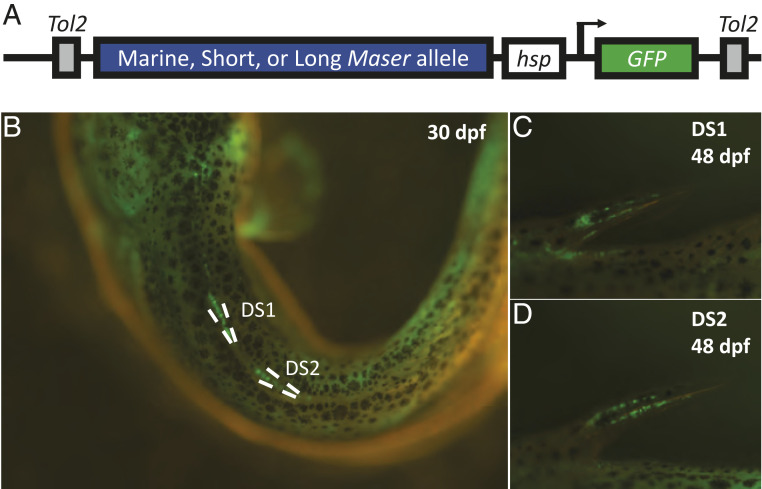
Transgenic expression reveals *Maser* activity in developing spines. (*A*) Overview of the injected constructs. (*B*) Earlier spine expression (30 dpf) was concentrated along the dorsal midline, especially at the base of the spines (20× magnification). (*C* and *D*) At slightly later time points, the expression becomes more concentrated in the spines themselves (lateral view, 40× magnification). Significant differences between the three constructs were not observed at any time point.

*Maser* maps in an intergenic region and so may contain important regulatory DNA sequences. To test for potential enhancer activity, we cloned the Long, Short, and Marine *Maser* alleles upstream of a *GFP* reporter gene and injected the three reporter constructs separately into fertilized stickleback embryos (Fig. 3*A*). The basal vector alone drives GFP expression in the lens of the eye (35), allowing for identification of successful transgenics by hatching. All three constructs drove consistent expression in the developing pectoral fins (PF), PS, and DS (Fig. 3 *B*–*D*). Clear GFP expression appeared in the PF at 10 d postfertilization (dpf), in the emerging dorsal spines at 20 dpf, and in the emerging pelvic spines at 30 dpf. Within the spines, expression was observed along the length of the spine and at the base (Fig. 3 *C* and *D*). Expression levels peaked in all three tissues around 35 dpf, or 15-mm standard body length, before slowly declining over the following 60 d. While there were no obvious qualitative differences in the expression patterns driven by the three different constructs, we note that quantitative changes in expression levels would be difficult to detect by fluorescent microscopy in transgenic fish.

To look for quantitative effects of the *Maser* region on the expression of endogenous stickleback genes, we used RNA-sequencing (RNA-seq) of larvae generated by crossing fish with contrasting *Maser* alleles. An unfertilized clutch of eggs from a representative Marine population (Rabbit Slough) was divided in half and fertilized by sperm from either a Long spine population (Mayer Lake) or a Short spine population (Bear Paw Lake). At 15-mm standard body length, we dissected developing tissues from the F1 hybrid larvae, and prepared RNA-seq libraries for 12 sets of PS, 12 sets of DS, 6 pairs of PF, and 6 hearts from each cross. We generated an average of 14.5 M 150-bp paired-end Illumina reads for each library and aligned to *gasAcu1-4* with STAR two-pass mapping (36).

We hypothesized that a gene contributing to divergent spine morphology would likely exhibit reciprocal allele-specific expression (ASE) differences between the two crosses, such as increased freshwater expression relative to marine in the Long spine cross and decreased freshwater expression relative to marine in the Short spine cross, or vice versa. We further hypothesized that this differential ASE would likely be tissue-specific, as in other known examples of key regulatory changes underlying stickleback evolutionary traits (15, 24, 37).

**Fig. 4.**
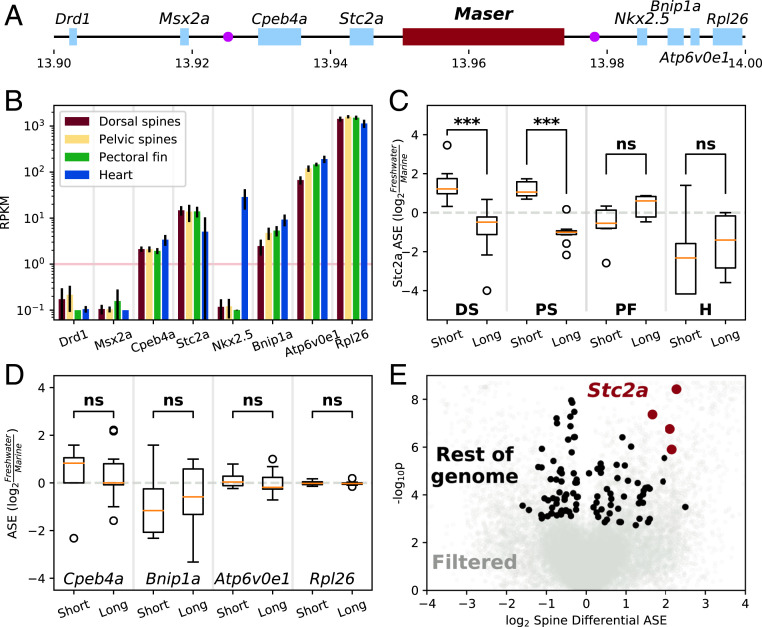
RNA analysis identifies *Stc2a* as target of *Maser*. (*A*) Genomic context around *Maser* (red). Light blue rectangles represent nearby genes, while the two purple circles are the top QTL markers for spine length (Stn235, *Left*, and Stn238, *Right*) (25). (*B*) Expression levels of all nearby genes in four different tissues. The horizontal pink line marks the approximate threshold of our ability to analyze expression at this sequencing depth. Error bars are SD. (*C*) ASE of *Stc2a* at chrIV:13943514 between Marine × Short (Short) and Marine × Long (Long). Significant differences between the Short cross and the Long cross are observed only in the DS and PS (***P < 0.001), and trend in the opposite direction in both control tissues, PF, and heart. (*D*) Dorsal spine ASE of other genes in the region. Results are similar for all genes in all other tissues. (*E*) All genome-wide SNPs analyzed for reciprocal differences in ASE in dorsal spines and pelvic spines between the Short and Long crosses. SNPs with weak or discordant ASE in spines or significant ASE differences in control tissues are in light gray (*Materials and Methods*). SNPs within *Stc2a* that pass these filters are highlighted in red.

*Maser* is located in a gene-rich region near multiple candidate loci with plausible roles in spine development (Fig. 4*A*), including: *Msx2a*, a homeobox-containing transcription factor with known roles in osteoblast differentiation and skeletal development (38, 39) and previously shown to undergo differential splicing between marine and freshwater stickleback (25); *Stanniocalcin2a*, a secreted glycoprotein with autocrine or paracrine functions and a potent inhibitor of bone growth (40); and *Nkx2.5* (*tinman* in *Drosophila*), a homeobox-containing transcription factor canonically associated with heart development but recently shown to be co-opted during emu limb development (41). Of these three candidate genes, our RNA-seq data showed that only *Stanniocalcin2a* (*Stc2a*) was expressed at appreciable levels in the dorsal and pelvic spines at this developmental stage (Fig. 4*B*).

*Stc2a* also fulfilled our other hypotheses by exhibiting reciprocal ASE differences between the two crosses in the pelvic spines and dorsal spines, but not in the PF or heart (Fig. 4*C*). Specifically, after quantifying ASE with the GATK tool ASEReadCounter (42), we find that in both the dorsal and pelvic spines the Short allele is up-regulated 2.7-fold relative to Marine, while the Long allele is down-regulated 1.8-fold relative to Marine (Dataset S7). This represents a fivefold increase in expression of the Short allele relative to the Long allele. The direction of change in the two alleles is consistent with the literature reports of *Stc2a* functioning as an inhibitor of bone growth. This difference is highly statistically significant by both parametric and nonparametric tests (*P* = 3.74e-9 by Mann–Whitney *U* test, *P* = 1.65e-28 by Fisher’s exact test for chrIV:13943514). In contrast, in both control tissues, there is no significant difference between the two freshwater alleles and the trend is actually in the opposite direction, with slightly higher expression from the Long allele relative to the Short.

None of the other genes near *Maser* display this reciprocal ASE (Fig. 4*D*). In fact, the four SNPs in the entire genome-wide dataset that best match our hypotheses for a gene controlling spine length are all located within *Stc2a* (Fig. 4*E*). These SNPs are distributed across the transcript and so do not reflect differential splicing (*SI Appendix*, Fig. S6*A*). There are no amino acid differences between the Long and Short *Stc2a* transcripts, although there are two nonsynonymous changes between Marine and both freshwater alleles (*SI Appendix*, Fig. S6*B*).

Previous research suggests that *Stc2a* acts by inhibiting PAPPA-mediated proteolytic release of bioactive IGF1 from its inactive state bound to IGFBP4 (43). Gene ontology (GO) analysis of genes differentially expressed between these two crosses yields significant enrichments for terms consistent with IGF pathway activation, including insulin receptor binding, calcium ion binding, gluconeogenesis, and skeletal system development (Dataset S8).

To further test the model that *Stc2a* activity influences stickleback spine lengths, we injected fertilized stickleback embryos with Cas9 protein and CRISPR guides targeting *Stc2a* exons. The injections induced a variety of mutations predicted to decrease *Stc2a* function, and mosaic founder fish showed significant increases in the length of DS1, DS2, and the PS (*SI Appendix*, Fig. S7). No significant changes in length were seen for the smaller third dorsal and anal spines, consistent with the reduced influence of *Maser* genotypes on these smaller spines in association studies (*SI Appendix*, Fig. S4).

### Recombination Rates in the *Eda*-*Stc2a* Region.

**Fig. 5.**
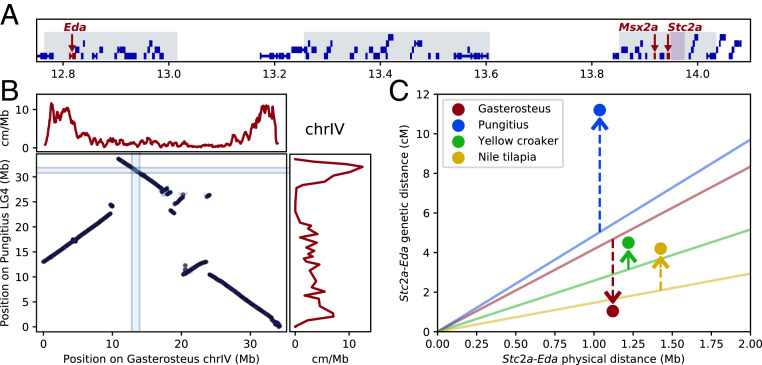
The spine and armor plate supergene and other repeatedly differentiated regions feature reduction in recombination rate in threespine stickleback. (*A*) An overview of the spine and armor plate supergene region on chrIV (scale in megabases). Most genes are in blue and genes linked to armor and spine changes are in red. Repeatedly differentiated regions are shaded gray and *Maser* is shaded light purple. (*B*) Comparison of synteny and recombination rates between the threespine stickleback (*Gasterosteus*) and its close relative the ninespine stickleback (*Pungitius*). The light blue bars denote the location of the spine and armor supergene in both species (*Eda* to *Stc2a*). (*C*) A comparison across *Gasterosteus*, *Pungitius*, and other outgroup species of the genetic and physical sizes of the *Eda*-*Stc2a* interval (denoted by circles), and whether recombination in this interval is increased or decreased (arrows) relative to the average genome-wide recombination rate observed in each species (solid lines). Relatively decreased *Eda2-Stc2a* recombination is seen in *Gasterosteus* but not the outgroup species.

The *Maser*-*Stc2a* region maps only 1.1 Mb from the major locus controlling armor plate differences in threespine stickleback (*Eda*) (Fig. 5*A*) (14). This interval contains many ecotypically differentiated genomic sequences, and overlaps multiple major QTL that control armor, feeding, and behavioral differences mapped in genetic crosses in a “supergene” complex (44, 45). As tight genetic linkage between multiple complementary but distinct loci and traits is a defining characteristic of “supergenes” (46), we compared recombination rates and synteny in threespine stickleback (*Gasterosteus aculeatus*) and closely related ninespine stickleback (*Pungitius pungitius*), with yellow croaker (*Larimichthys crocea*) and Nile tilapia (*Oreochromis niloticus*) as additional outgroups. Local gene order is conserved between *Gasterosteus* and *Pungitius* in the *Eda*-*Stc2a* region. However, threespine stickleback show a striking decrease in genetic recombination rates in this region compared to ninespine stickleback (Fig. 5*B*) (47), yielding a local recombination rate substantially below the genome-wide average in threespine stickleback (Fig. 5*C*). In contrast, ninespine stickleback and both outgroup species show higher recombination rates in the *Eda*-*Stc2a* interval compared to genome-wide averages (Fig. 5*C*) (48, 49), suggesting the lowered recombination rate is a derived trait in *Gasterosteus*.

### Genomic Survey for Triallelic Differentiation in Stickleback.

**Fig. 6.**
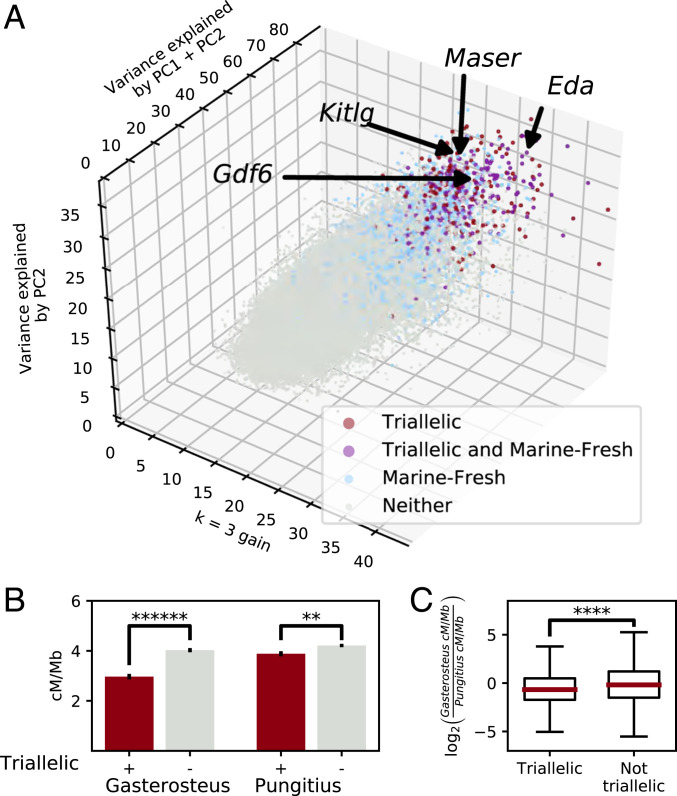
Triallelism is a common phenomenon. (*A*) Visualization of the multiple axes of variation in 2,500-bp windows across the genome. *Maser*, *Eda*, *Kitlg*, and *Gdf6* are typical of triallelic regions, with substantial variance explained in both of the first two principal components (PC1 and PC2), and improved clustering with three rather than two clusters (*k* = 3 gain) (*SI Appendix*, Fig. S2). (*B*) Tiled 100-kb windows that overlap triallelic regions (red) have significantly lower recombination rates than nontriallelic windows (gray) in *Gasterosteus* (******P = 1.88e-11), while the syntenic regions in *Pungitius* only differ slightly (**P = 0.011). Error bars are SEM. (*C*) Triallelic regions across the genome have lower recombination rates in *Gasterosteus* (relative to *Pungitius*) than nontriallelic regions (ratio of values in *B*, ****P = 1.01e-4).

Several previous studies have identified genomic sequences that show prominent differentiation between most marine and freshwater stickleback (16–18, 50, 51). Having identified multiple alleles at *Maser* that are associated with contrasting phenotypes among different freshwater populations, we searched stickleback genome sequences for other loci that also show triallelic differentiation among diverse populations from the Pacific Northwest. We hypothesized that other triallelic regions of the genome would share two key properties we observe in *Maser*: significant variation along multiple axes (not just a single marine versus freshwater axis) and clustering of populations into three groups (not two, as expected for simple marine–freshwater differentiation). To test this, we used PCA to perform dimension reduction on sequence data from 69 populations in 2.5-kb windows tiled across the genome. We then filtered for multiple axes of variation (substantial variation along both PC1 and PC2), and improved clustering scores with three rather than two clusters (*Materials and Methods*). This analysis identifies 494 regions with triallelic properties similar to *Maser* (median size 5 kb, 4.31 Mb total, 0.9% of genome) (Fig. 6 and Datasets S2 and S3).

Triallelic loci typically have a single allele in marine populations and two or more freshwater alleles that are not readily split by geography. Many triallelic modules overlap regions of consistent marine–freshwater differentiation [15 of 84 overlaps observed vs. 1 of 84 expected with 2% false-discovery rate loci (17), *P* < 0.00001], including previously characterized loci regulating armor plate development, *Eda* (14) and *Gdf6* (33), and gill and body pigmentation, *Kitlg* (15) (*SI Appendix*, Fig. S2 and Dataset S11). Some triallelic loci exhibit significantly greater divergence between contrasting freshwater alleles than between the marine and freshwater alleles (*SI Appendix*, Fig. S2), indicating that the primary axis of differentiation is associated with variation among freshwater habitats rather than general marine–freshwater ecotypic differentiation. As at *Maser*, triallelic loci are associated with regions of reduced recombination in the genome (Fig. 6*B*). Similarly, these reductions are likely to be derived in *Gasterosteus*, with syntenic loci in *Pungitius* showing smaller differences in recombination rate (Fig. 6 *B* and *C*).

Triallelic regions are strongly enriched for genes with reciprocal changes in freshwater expression in our RNA-seq data (8 of 36 observed vs. 3 of 36 expected within 50 kb of triallelic region, *P* = 0.0088). These reciprocally expressed genes are also strongly enriched for a variety of immune categories including both class I and class II MHC, interleukin and cytokine production, and response to bacteria, viruses, and fungi that are likely to vary substantially among stickleback environments (Dataset S10) (52).

## Discussion

Threespine stickleback are named for their prominent dorsal spines and pelvic apparatus (*Gasterosteus*: bony stomach; *aculeatus*: prickly thorns). Elevation and locking of the dorsal and pelvic spines substantially increases the cross-sectional area of stickleback and helps protect body tissues against compressive and twisting forces by gape-limited avian and fish predators (53, 54). Conversely, short spines and reduced supporting armor may increase body flexibility and minimize grasping surfaces used by macroinvertebrate predators (55). Ecological correlations and experimental tests suggest that long and short spines are favored by different predation regimes in different environments (53, 56–60). Here we identify a genetic mechanism that can explain both increases and decreases in spine length in stickleback populations, through multiple ancient alleles in a major spine enhancer region located immediately upstream of *Stanniocalcin2a*.

Previous studies confirm that stanniocalcin is a bidirectional modulator of growth control in many species. *Stc2* knockout mice are 10 to 15% larger and grow faster than wild-type mice (61). Conversely, transgenic mice overexpressing *Stc2* are 45% smaller (40). In humans, an exome-wide search for coding variants affecting height found the single largest variant to be a rare missense mutation reducing *Stc2* activity, resulting in a 2.1-cm height increase in heterozygous carriers (62). Finally, one of the strongest predictors of overall size among dog breeds is a SNP 20 kb downstream of *Stc2*, where small dog breeds nearly universally feature the derived allele (63). While this allele has not been functionally characterized, it likely acts as a *cis*-regulatory element to increase expression of *Stc2* in a manner similar to *Maser.*

Genes with bidirectional “hypomorphic” and “hypermorphic” alleles have long intrigued biologists (64, 65), and may be particularly good substrates for evolutionary change. Mechanistic studies have shown that STC2 acts by modulating the local availability of the growth factor IGF1, by serving as a competitive inhibitor of a protease that is required to release IGF1 from the binding protein IGFBP4 (43). The direction of effects we see in stickleback are completely consistent with this mechanism. The Short spine *Maser* allele increases stanniocalcin expression in developing spines (predicted to lead to decreased proteolysis of IGBP4 and decreased IGF1 activity), while the Long spine allele decreases stanniocalcin expression (predicted to lead to increased proteolysis of IGBP4 and increased IGF1 activity). Similarly, CRISPR-mediated loss of *Stc2a* increases stickleback spine lengths, matching the direction expected from both our RNA expression data and the mammalian literature. Notably, stanniocalcin acts as a local rather than global modulator of IGF1 activity in previous studies, changing growth in particular tissues without altering circulating levels of IGF1 hormone in the bloodstream (40). Similarly, *Maser* acts as a spine-specific enhancer of the *stanniocalcin* gene (Figs. 3 and 4), providing an elegant mechanism for either increasing or decreasing the local growth of dorsal and pelvic spines relative to overall body size.

Spines work together with underlying basal bones, armor plates, and pelvic structures to make a bony girdle surrounding the middle of the stickleback body (54). Strikingly, the major genes controlling different aspects of this bony girdle are tightly linked with one another in a supergene complex on chrIV (25, 44), including *Eda*, the major armor plate locus (14), and *Msx2a*, a gene associated with reduced dorsal but not pelvic spine lengths in many freshwater fish (25). Supergenes are clusters of linked but separable functional genetic elements that control alternative complex phenotypes in populations (46). Molecular studies show supergenes may consist either of multiple regulatory elements controlling a single developmental gene, tandem duplications of structurally related genes, or aggregates of structurally diverse genes that nonetheless influence traits that have related functions. Identification of *Maser* and *Stanniocalcin2a* adds to emerging evidence that a stickleback supergene complex on chrIV consists of multiple distinct genes and regulatory sequences contributing to linked, ecologically important traits.

Low recombination rates across the *Eda-Stc2a* supergene region appears to be a derived trait in *Gasterosteus*, with substantially higher recombination rates seen across the same interval in ninespine stickleback and other fishes. Local gene order is preserved in the *Eda-Stc2a* interval between *Gasterosteus* and *Pungitius*, suggesting the supergene has not been assembled by duplications or translocation of new genes within the region. However, large-scale rearrangements place the *Eda-Stc2a* region in the middle of chrIV in *Gasterosteus*, where recombination rates are typically lower than at chromosome ends, where they are located in *Pungitius* (Fig. 5*B*). Further studies will be required to determine the mechanisms that contribute to reduced recombination rates. On a broader phylogenetic scale, we note that changes in the intergenic spacing of *Maser* and *Stc2a* have occurred in most spiny-rayed compared to soft-rayed fishes (*SI Appendix*, Fig. S8). Such spacing changes may have contributed to the emergence of the *Maser*-*Stanniocalcin* control mechanism in fishes with spiny fin rays, and to the overall evolutionary success of the spiny-rayed acanthomorpha, which make up 85% of all marine fish species (19, 20).

Many other regions of the stickleback genome show triallelic differentiation patterns similar to those we find in the *Maser* region. We speculate that these loci may represent additional cases in which the same underlying gene is being used to control multiple alternative phenotypes in different environments. Interestingly, triallelic loci include the *Eda*, *Gdf6*, and *Kitlg* genes, previously shown to be major loci controlling armor plate numbers, armor plate sizes, and body pigmentation, respectively (14, 15, 33). We note that many aspects of skeletal armor and pigmentation vary both within and among freshwater populations, including presence or absence of particular anterior plates, changes in the shapes and overlaps of bony plates in populations with different spine phenotypes, and changes in body pigmentation in environments with different water colors, competitors, and predators (54, 60, 66–69). Triallelic differentiation is also seen at many stickleback immune loci, some of which also show evidence of reciprocal up- and down-regulation in our genome-wide expression surveys of different stickleback crosses. A key challenge for the future will be to determine whether different alleles at these loci also control contrasting morphological, physiological, or immunological phenotypes.

Classic multiallelic polymorphic loci are known in other organisms, often associated with host–pathogen interactions, or maintaining multiple defensive, sensory, or reproductive strategies within a species (70–74). As in sticklebacks, additional multiallelic loci are currently being uncovered as large-scale sequencing and trait association studies localize multiple genotypes and phenotypes to particular loci in the genome (75–77). Interestingly, some of the best-characterized multiallelic polymorphisms in other organisms also appear to be ancient polymorphisms whose alternative alleles evolved long before the recent populations or species in which they are currently found (71, 73, 75). Diversification of ancient standing variation into multiple specialized alleles may provide a common mechanism for adapting to a range of conditions, making it possible to evolve many different phenotypes from a smaller toolkit of key developmental loci.

## Materials and Methods

### Initial Identification of *Maser.*

Fish collected from around the world were measured with digital calipers for length of DS1, DS2, right PS, and standard body length (Dataset S1). Missing or broken spines were excluded from all analysis. After regression on standard body length, fish were divided into quintiles for each spine and the first and last quintiles were compared at SNPs across the genome. The associated genome sequences are available at the Sequence Read Archive (accession no. PRJNA247503) and are more thoroughly described by Marques et al. (50) and Roberts Kingman et al. (18).

### PCR-Based Fine-Mapping Genotyping.

From the cloned and sequenced Mayer Lake and Bear Paw *Maser* alleles, 13 markers were designed to yield different-sized bands on an agarose gel either immediately after PCR due to large structural variations or after restriction digest at a SNP differing between the two alleles. DNA was prepared for PCR analysis by phenol-chloroform extraction for all samples. All samples were analyzed at all markers and sexed by *IDH* genotyping as previously reported (78). See Datasets S4 and S9 for primer sequences, PCR information, and restriction conditions.

### Fine-Mapping Analysis.

Samples for association mapping were collected on 1 June 2019 from Matanuska Lake (61.553°N, 149.228°W and 61.554°N, 149.226°W) and on 2 June 2019 from Little Meadow Creek (61.569°N, 149.569°W), anesthetized in Tricaine (Western Chemical, MS-222), and preserved in 90% ethanol. Regression was performed against standard body length and sex. Sex is significant in Matanuska Lake but not Little Meadow Creek. In the main text, 2007 and 2019 samples were regressed separately and then pooled and analyzed jointly for clarity. The two time points do not differ from each other and the results are similar if they are instead analyzed separately. Genotype-phenotype association was tested at each marker by *F*-test.

### Reference Genome.

All coordinates and alignments are relative to reference genome version *gasAcu1-4* (18). See also https://datadryad.org/stash/dataset/doi:10.5061/dryad.547d7wm6t for further resources on this reference version.

### Whole-Genome Association Mapping.

After regression on standard body length, 24 fish with long spines and 24 with short spines were selected for sequencing from both Little Meadow Creek and Matanuska Lake 2019 samples, for a total of 96 fish. An equal number of males and females were sequenced from Little Meadow Creek, but only males were sequenced from Matanuska Lake due to the observed sex effects in that population. Phenol-chloroform extracted DNA samples were further purified through the PureLink Genomic DNA Minikit (ThermoFisher #K182001). Libraries were prepared with the Illumina Nextera DNA Flex kit with Unique Dual Indexes following the manufacturer’s instructions (Illumina #20018705, Illumina #20027213) and sequenced across four HiSeq 4000 lanes by the Novogene Corporation, yielding 1,818 M 150-bp paired-end reads (12× average coverage per sample). The reads were aligned to *gasAcu1-4* using bwa mem and variants called with GATK following its Best Practices. The called variants were then tested for association with phenotype as described in the main text.

### RNA-Seq.

One-half of a clutch of unfertilized eggs from a female Marine stickleback (Rabbit Slough) was fertilized by frozen sperm from a Short freshwater population (Bear Paw) and the other half fertilized by frozen sperm from a Long freshwater population (Mayer Lake). At 35 dpf and 15-mm standard body length, fish were killed in Tricaine (Syndel, MS-222) and dissected, alternating between crosses. The following tissues were dissected: DS1 and DS2, in a single piece, including tissue between and just underneath the spines; PS, in a single piece, including the pelvic girdle but not the ascending process, and extremely minimal soft tissue; PF, left and right, cut at their base and pooled; and heart (H).

After dissection, tissues were immediately placed in RNAlater (ThermoFisher AM720) and stored at 4 °C overnight. The next morning, samples were homogenized by FastPrep (0.25-inch ceramic spheres, MP #6540424, for PF, DS, and PS; Lysing Matrix D, MP#116913050 for H), RNA extracted with the NucleoSpin RNA XS kit (Takara #740902.5), and RNA stored at −80 °C until library preparation. Libraries were prepared using the TruSeq Stranded mRNA Library Prep kit with Unique Dual Indexes (Illumina #20020595, Illumina #20022371), following the manufacturer’s instructions. For each cross, 12 DS libraries, 12 PS libraries, 6 PF libraries, and 6 H libraries were prepared, for a total of 72 RNA-seq libraries. Due to differences in RNA yields between tissue types, the following RNA input and PCR cycle counts were used: DS, 1,000 ng RNA, 10 cycles; PS, 500 ng RNA, 10 cycles; PF, 500 ng RNA, 10 cycles; H, 50 ng RNA, 15 cycles. Samples from both crosses were interspersed at all stages of RNA prep and library prep. Libraries were sequenced across two HiSeq 4000 lanes by the Novogene Corporation, yielding a total of 1,011 M 150-bp paired end reads. One Long PS sample failed completely and one Short PS sample yielded under 1 M reads; the other 70 libraries have a median of 14.5 M reads.

Reads were aligned against *gasAcu1-4* using STAR two-pass mapping (36) guided by lifted Ensembl gene annotations. Base quality was adjusted and variants called using GATK following its Best Practices. Shared variants for analysis were identified by selecting bases where at least 20 of 24 fish have at least three reads for each allele (minimum 5% of total reads), yielding 27,048 SNPs suitable for differential ASE analysis. At these sites, GATK ASEReadCounter was used to retrieve read count for each allele in each library.

### RNA-Seq Differential ASE.

Each of the 27,048 SNPs was analyzed by two separate methods for ASE between the two crosses in each of the four tissues.

First, we summed the reference reads and alternate reads within each cross to create a 2 × 2 contingency table, which we analyzed with Fisher’s exact test. Although we did not observe instances of more deeply sequenced samples biasing the results, we down-sampled to the median read depth for each cross and tissue to preclude this possibility.

Second, we took the log_2_ ratio of reference reads to alternate reads within each sample, and then compared the log ratios of samples from the Marine × Long cross to those from the Marine × Short cross with the Mann–Whitney *U* test. This combination of using read count only to calculate allelic ratios with a nonparametric test yields highly conservative *P* values.

### Spine Differential ASE Filter Criteria.

To identify SNPs behaving as expected for a gene controlling spine length, we used the following criteria: located in an annotated exon; not extreme sequencing depth outlier (<5,000 reads); DS and PS must both have significant differential ASE in the same direction (Mann–Whitney *U* unadjusted *P* < 0.05 for both tissues); no differential ASE in either control tissue (*P* > 0.05 and −1 < differential ASE < 1). SNPs failing any of these criteria are colored in light gray in Fig. 4*E*.

### Identification of Genes with Reciprocal Expression Patterns and Overlap with Triallelic Genomic Regions.

To identify SNPs with reciprocal expression patterns in at least one tissue, regardless of relevance to spine development, we used the following criteria: located in an annotated exon; not extreme sequencing depth outlier (<5,000 reads); and in at least one tissue, both freshwater alleles are significantly differentially expressed relative to marine (by binomial test) and to each other (by both Mann–Whitney *U* and Fisher’s exact test, as above), and have at least a twofold change in expression between the two freshwater alleles. This yielded 220 SNPs in 134 genes, or 122 SNPs in 36 genes if requiring support from at least 2 SNPs per gene. All five SNPs in *Stc2a* pass the above filters.

To test for enrichment near triallelic genomic regions that could be differentially regulating their expression, we counted the number of these reciprocal expression genes within 50 kb of the triallelic genomic regions and compared against a reference distribution of 10,000 sets of randomly selected genes. We found a statistically significant enrichment in the set of genes supported by at least two SNPs (*P* = 0.0088) but not when only requiring support from a single SNP (*P* = 0.14). Both stringencies yielded similar GO enrichments (Dataset S10).

### CRISPR Targeting and Fish Phenotyping.

Six single-guide RNAs targeting *Stc2a* were designed and synthesized as previously described (79), four targeting exon 1 and two targeting exon 4 (Dataset S9). Injection mixes were prepared with equal proportions of all six guides (300 ng/μL total concentration), 40 μM Cas9-nls protein (QB3 MacroLab, University of California, Berkeley), and 0.5% Phenol Red in 10 mM Tris⋅HCl pH7.5. Two clutches of fertilized stickleback embryos from Rabbit Slough were divided to produce roughly equal numbers of uninjected controls and surviving injected embryos.

Six months postfertilization, fish were anesthetized in tricaine (MS-222, one-third normal concentration, 0.1 g/L) and live-imaged by X-ray on a Faxitron UltraFocus X-ray cabinet. X-ray images were anonymized and randomized, then measured for length of DS1, DS2, DS3, anal spine, and PS, as well as standard length. Fish over 40-mm standard length were analyzed in *SI Appendix*, Fig. S7. Both clutches showed similar spine length patterns. All measurements can be found in Dataset S12.

### CRISPR Genotyping.

To confirm the efficacy of the CRISPR injections, DNA was collected from all injected and control fish by skin swab (80), followed by phenol-chloroform purification. Small amplicons were PCR-amplified with primers oGK1111/oGK1112 (exon 1) and oGK1115/oGK1116 (exon 4). Smeary bands suggestive of highly mosaic frameshift mutations were observed by agarose gel in approximately half of CRISPR-injected fish but no control fish. We performed TOPO cloning (ThermoFisher K4575-01) and Sanger-sequencing of individual colonies from several fish and confirmed the presence of a variety of genetic lesions at single-guide RNA target sites in both exons, including frameshift mutations, premature stop codons, and splice site alteration. However, as not all injected fish showed evidence of mutations, and the specific mutations and overall degree of mosaicism likely vary across tissues and individuals (79), the results presented in *SI Appendix*, Fig. S7 should be interpreted as a conservative lower-bound for the phenotypic effects of targeting *Stc2a* in stickleback.

### Triallelic Region Identification.

The genome was divided into overlapping 2,500-bp windows with a 500-bp step, and each window was analyzed by two methods. First, PCA was performed and the weighting of each axis was calculated. Windows with PC2 ≥ 20 PVE and PC1 + PC2 ≥ 75 PVE were selected as candidates, and any such windows within 10 kb were merged. Second, *k*-means clustering was performed with *k* = 2 and *k* = 3, and the sum *s* of average intracluster genetic distances was calculated. Windows with >65% reduction in *s* for *k* = 3 and with >20% reduction resulting from the transition from *k* = 2 to *k* = 3 were selected as candidates, and any such windows within 5 kb were merged. These parameters were chosen based on qualitative inspection to yield genomic regions approximately as triallelic as *Maser* or more so. The intersect of both methods was taken for the final triallelic call set (Dataset S2), which includes 494 regions spanning 4.3 Mb (0.93% of genome).

### Animal Care.

All animal studies were performed in accordance with the recommendations in the Guide for the Care and Use of Laboratory Animals of the National Institutes of Health (81). Stickleback experiments were performed using protocols approved by the Institutional Animal Care and Use Committee of Stanford University (IACUC protocol #13834), in animal facilities accredited by the Association for Assessment and Accreditation of Laboratory Animal Care International (AAALAC). Stickleback collected from wild populations were sampled under collecting permits from the Alaska Department of Fish and Game and killed under protocols approved by the Institutional Animal Care and Use Committee of Stony Brook University (IACUC protocol #2019-1354).

## Supplementary Material

Supplementary File

Supplementary File

Supplementary File

Supplementary File

Supplementary File

Supplementary File

Supplementary File

Supplementary File

Supplementary File

Supplementary File

Supplementary File

Supplementary File

Supplementary File

## Data Availability

All data supporting the findings of this study are included in the main text and *SI Appendix* or deposited in publicly available databases. All whole-genome sequencing data from Little Meadow Creek and Matanuska Lake are available at the Sequence Read Archive, https://www.ncbi.nlm.nih.gov/sra (BioProject PRJNA681039) (82). All RNA-seq data are available at the Sequence Read Archive (BioProject PRJNA681136) (83). The Sanger-derived *Maser* sequences have been submitted to GenBank (accession nos. MW308125 (84), MW308126 (85), and MW308127 (86) for Long, Marine, and Short, respectively). Sequencing data from Marques et al. 2018 (50) and Roberts Kingman et al. 2021 (18) used here are available at the Sequence Read Archive (BioProject PRJNA247503) (87). Any materials will be made available upon request.
