# Supplementary material for: Longer or shorter spines: Reciprocal trait evolution in stickleback via triallelic regulatory changes in Stanniocalcin2a
Source: Proc Natl Acad Sci U S A. 2021 Jul 28;118(31):e2100694118. doi: 10.1073/pnas.2100694118 (PMC8346906; doi:10.1073/pnas.2100694118)
Supplement: Supplementary File [file pnas.2100694118.sapp.pdf]

PNAS
www.pnas.org

Supplementary Information for

Longer or shorter spines: reciprocal trait evolution in stickleback via triallelic regulatory changes in *Stanniocalcin2a*

Garrett A. Roberts Kingman, David Lee, Felicity C. Jones, Danielle Desmet, Michael A. Bell, and David M. Kingsley

Corresponding author: David M. Kingsley
Email: kingsley@stanford.edu

This PDF file includes:

Supplementary Methods
Figures S1 to S8
Legends for Datasets S1 to S12
SI References

Other supplementary materials for this manuscript include the following:

Datasets S1 to S12

Supplementary Methods

Cloning and sequencing of *Maser*

Genomic DNA was prepared through phenol chloroform extraction of tail clips for fish from Rabbit Slough (Marine), Mayer Lake (Long), and Bear Paw Lake (Short). Fragments of *Maser* were then scarlessly introduced into the pT2HE vector (1) prepared with additional restriction sites and digested with *DpnI*. The genomic DNAs were then PCR amplified with oGK573/oGK521, vector and insert were digested with *SfiI* and *MluI*, ligated together, transformed, and colonies with the correct insert picked, grown up, and purified. This step was repeated three additional times, with primers oGK580/oGK581, oGK535/oGK582, and oGK882/oGK883, with digest by *MluI* and *AvrII*, *AvrII* and *SgrAI*, and *SgrAI* and *PvuI*, respectively. The three cloned alleles were then Sanger sequenced with overlapping reads (see Table S9 for primers). Pairwise sequence divergence rates were then calculated from SNPs (excluding structural variants and indels) and compared to divergence with the Japan Sea stickleback, which geological records indicate diverged ~2 Mya (2).

Principal component analysis

Following variant calling, we performed dimensionality reduction through principal component analysis (PCA). For each genomic window of interest containing N variable sites, we transformed genotype into an N-dimensional vector for each sample. We excluded sites where >1/3 of samples were missing genotype calls and imputed any missing data at other sites using a K-nearest neighbors algorithm (with k=3) on up to 100 neighboring sites in the genomic window. We performed PCA using the sklearn python library and extracted both the variance explained by each principal component axis and the coordinates of each sample along these axes using the inbuilt library methods.

Sample collection and measurements

Fish were collected from Little Meadow Creek and Matanuska Lake, euthanized in tricaine (MS-222, Syndel), immediately fixed in 70% ethanol, and measured for standard length, dorsal spine 1, dorsal spine 2, dorsal spine 3, anal spine, and the right pelvic spine. All fish collected in 2007 were measured in triplicate with digital calipers. Follow-up sampling from 2018 yielded an excess of fish, so fish with more visually extreme phenotypes were selected for measurement and genotyping. The 2018 fish had their right pelvic spine measured in triplicate with digital calipers and other measurements taken by X-ray, and later all verified by triplicate caliber measurement. All fish were regressed against standard body length and sex only within their sample cohort to control for any differences in measurement and effects of long-term ethanol storage.

Transgenic stickleback assays

Transgenic stickleback were generated as previously described (3), through microinjection of plasmids with *Tol2* transposase into single cell stickleback embryos from Matadero Creek, California. Larvae were anaesthetized with tricaine (MS-222, Syndel) and imaged for GFP expression approximately once a week throughout the first two months of development. Images were taken with a MZFLIII fluorescent microscope (Leica Microsystems, Bannockburn, IL) using GFP2 filters and a ProgResCF camera (Jenoptik AG, Jena, Germany).

Triallelic overlap significance

The triallelic regions were shuffled by bedtools, and the number of CSS regions (2% False Discovery Rate)(4) overlapping a shuffled triallelic region was counted across 100,000 simulations. No random permutations had as many overlaps as observed, so $p < 1e-5$ was taken as an upper bound.

Gene ontology

Stickleback genes were mapped to the corresponding human 1-to-1 orthologs (by Ensembl 94). Stickleback genes without 1-to-1 human orthologs were not used for gene ontology analysis. The associated tag counts were then evaluated by hypergeometric distribution and p-values adjusted for multiple comparisons by false discovery rate.

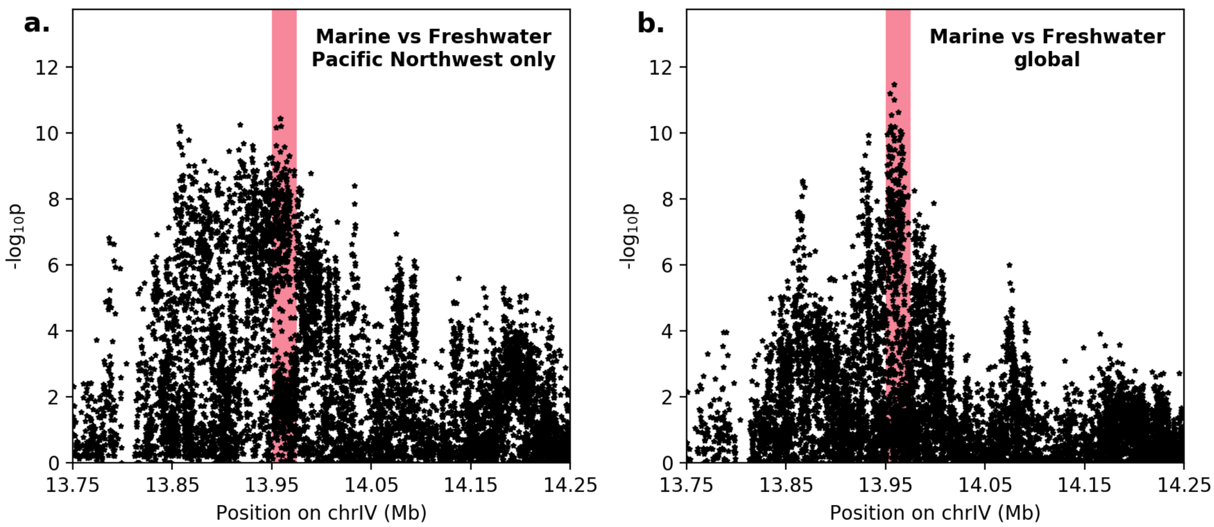

Fig. S1. Genotype-ecotype association in the genomic region around *Maser* (pink), in just the ancestral Pacific Northwest (a) or around the world (b). p-values are unadjusted and as previously reported (5). *Maser* is the peak of the globally shared genomic region, but only part of a much larger haplotype found across the Pacific Northwest.

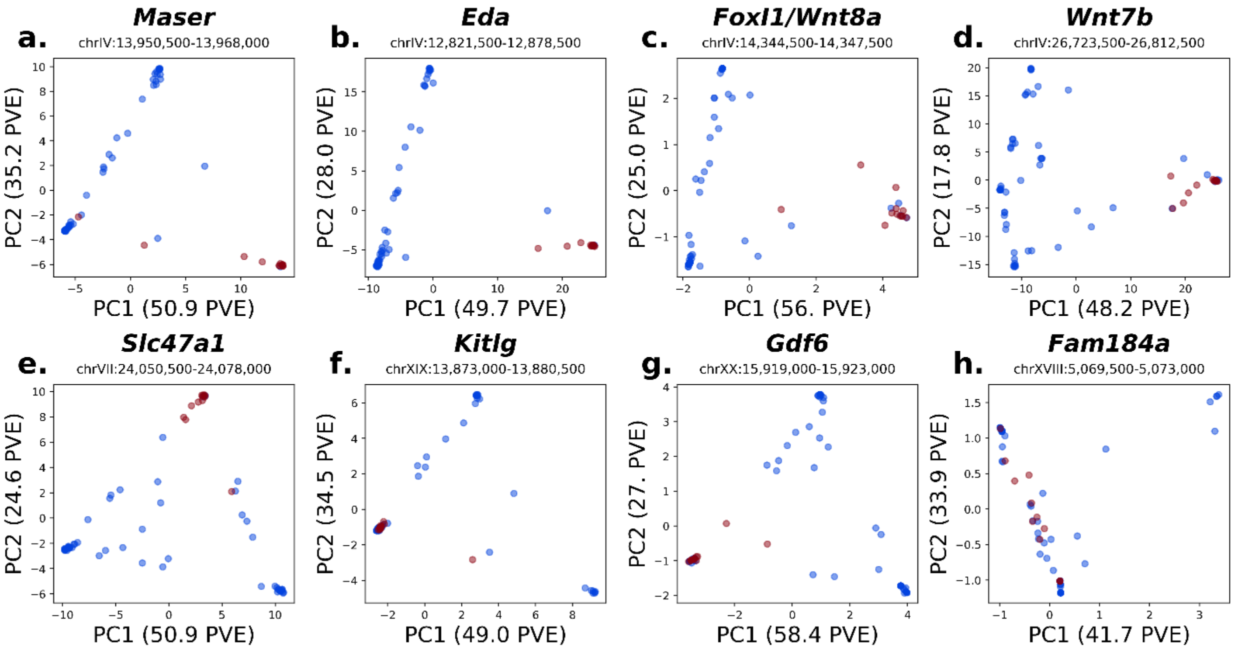

Fig. S2. Triallelic patterns observed at ecotypically differentiated stickleback loci. For all panels, blue dots represent freshwater populations and red dots represent marine populations in the Pacific Northwest, and the clusters do not represent obvious geographic proximity. In addition to the clustering of the plotted points, note how all panels have substantial loading on both the first and second principal component axes. a. *Maser* is intergenic and located between *Stc2a* and *Nkx2.5*. b. The triallelic locus at *Eda* (~1Mb upstream of *Maser*) overlaps the coding regions of *Eda*, *Tnsfs13b*, *Garp*, *cx31.7*, *Mtnr1c*, and *Neurl1b*, as well as the conserved 16 kb region associated with armor plating (6). c. This locus is only 400 kb downstream of *Maser* and located between *Foxl1* (5 kb upstream) and *Wnt8b* (two copies, located 1.4 kb and 3.6 kb downstream). d. A large (89 kb) triallelic region overlapping *Mirlet7a3*, *Mirlet7b*, *Pparaa*, *Cdpf1*, and 5.4 kb from *Wnt7b*. e. Overlapping a cluster of copies of *Slc47a1* and just downstream of *Vgfl1* (9 kb). f. 2.5 kb upstream of *Kitlg*, a major determinant of pigmentation in stickleback and humans (7). g. A smaller 4 kb region intronic to *Bckdhb*, and directly overlapping a previously characterized 4 kb enhancer of *Gdf6* that regulates armor plate development (8). h. An example of a triallelic region that does not involve marine-freshwater differentiation, overlapping *Fam184a*. Note how in several of these examples, the two major freshwater clusters are separated by PC1, while marine and freshwater are largely separated by PC2.

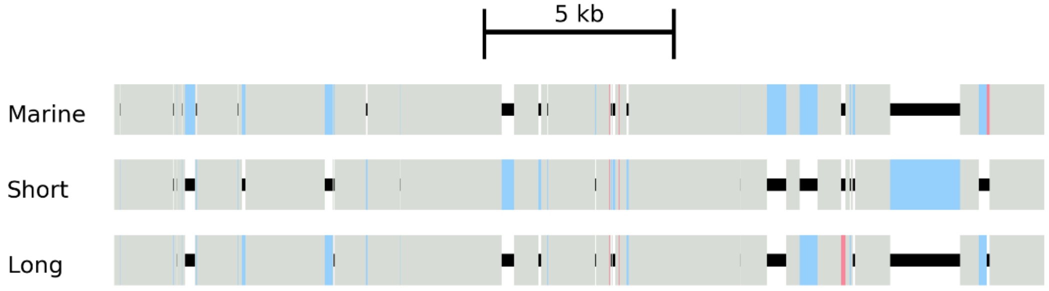

Fig. S3. Structural variation within *Maser*. Gaps are indicated by thin black lines, sequences aligned against gaps by light blue, and $(TG)_n$-dinucleotide repeats in pink.

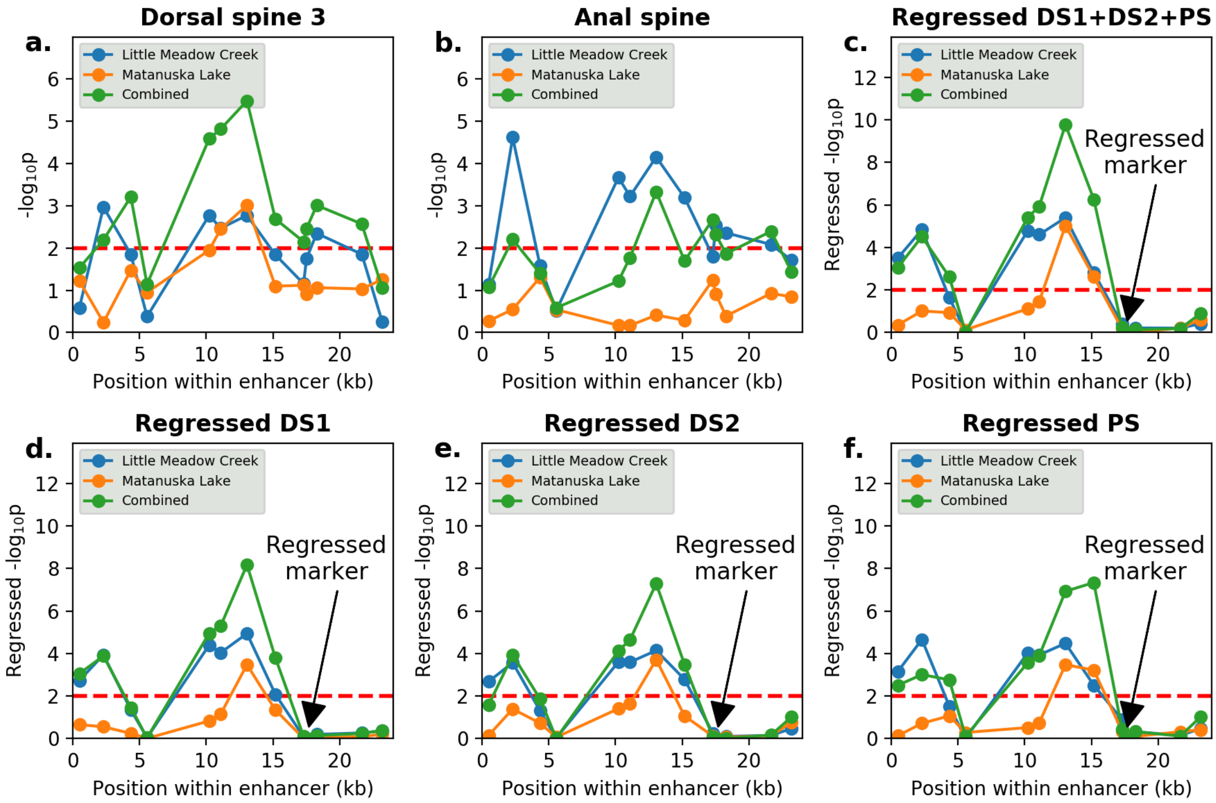

Fig. S4. Weaker association with length of dorsal spine 3 and anal spine, and signal at multiple linked markers. a, b. Association between genotype and length of either dorsal spine 3 (a) or anal spine (b) at 13 PCR-based markers tiled across *Maser*. The dashed red line represents an uncorrected p-value of 0.01. Both Little Meadow Creek and Matanuska Lake show association with length of dorsal spine 3, but only Little Meadow Creek shows association with anal spine length. c-f. Association of spine length residuals with genotype after controlling for genotype at the peak marker (indicated). The substantial remaining signal indicates they are not significant in the earlier plots only by linkage disequilibrium, but also contain additional information.

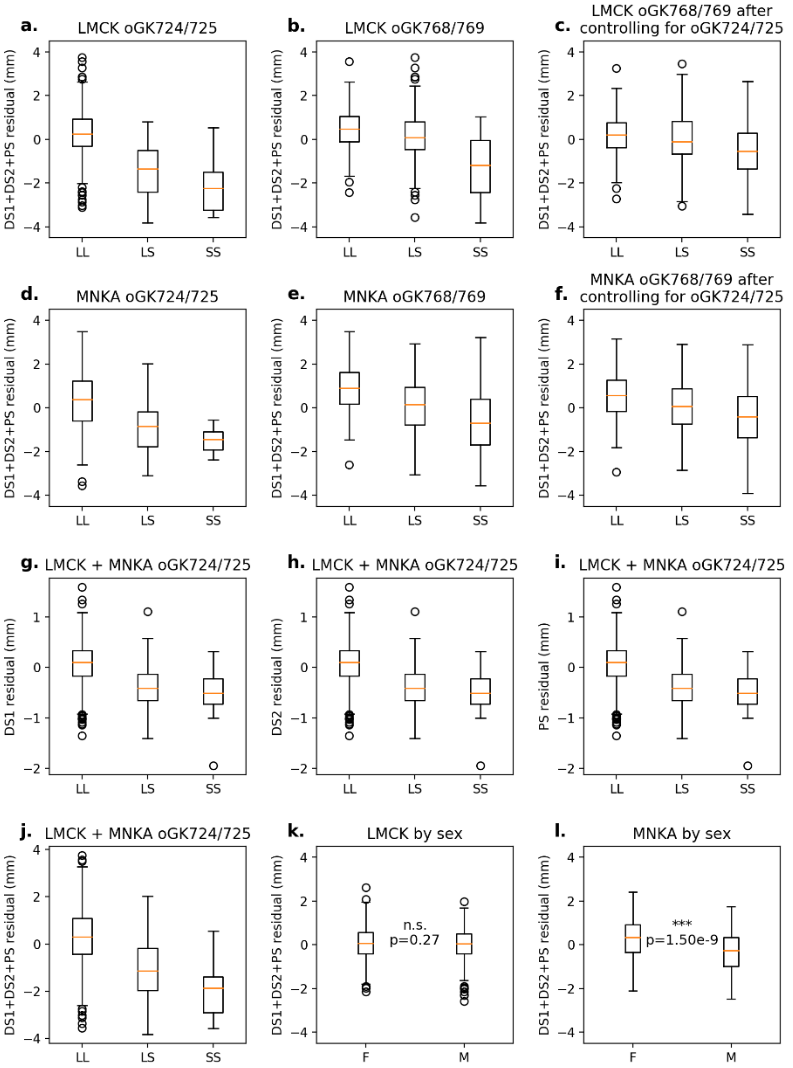

Fig. S5. Phenotypic stratification by population, trait, marker, and sex. a-j. Fish are stratified by genotype at the indicated marker in Little Meadow Creek (LMCK) and Matanuska Lake (MNKA). oGK724/725 is the primary peak marker and oGK768/769 is the secondary peak marker in Figure 3f. k-l. Fish are stratified by sex. Sex is removed from the phenotypic regression in panels k and l. All phenotypes are significantly associated with the genotype at the indicated markers except sex in Little Meadow Creek (see Figure 3). In this and other figures, the box plots show the median value (orange line) and 1st and 3rd quartiles, with whiskers extending to 1.5x the interquartile range.

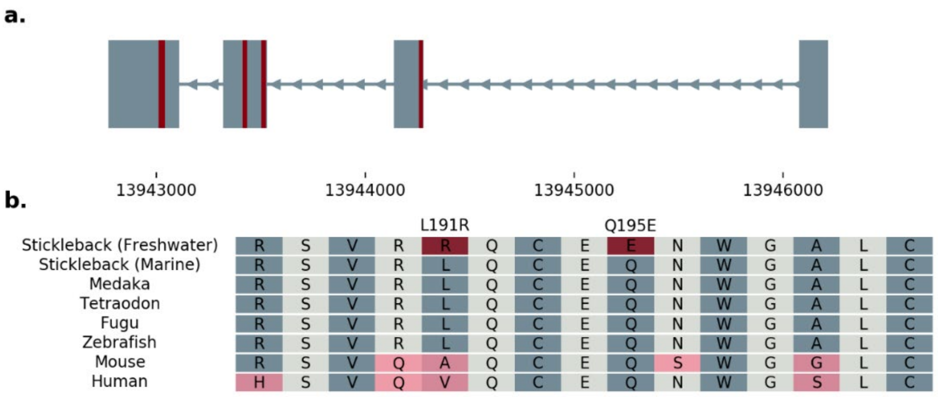

Fig. S6. Variants in the coding sequence of *Stc2a*. a. SNPs in *Stc2a* used in the allele-specific RNA-seq analysis. At the nucleotide positions shown, the Long and Short alleles share the same base, while the marine allele has a different base. Note that exon 4 contains two such SNPs 11bp apart that cannot be visually distinguished here. b. The two exon 4 SNPs result in amino acid changes in deeply conserved positions of *Stc2a*. The other 3/5 SNPs shared between Long and Short alleles and 2/2 SNPs that differ between Long and Short do not result in amino acid changes.

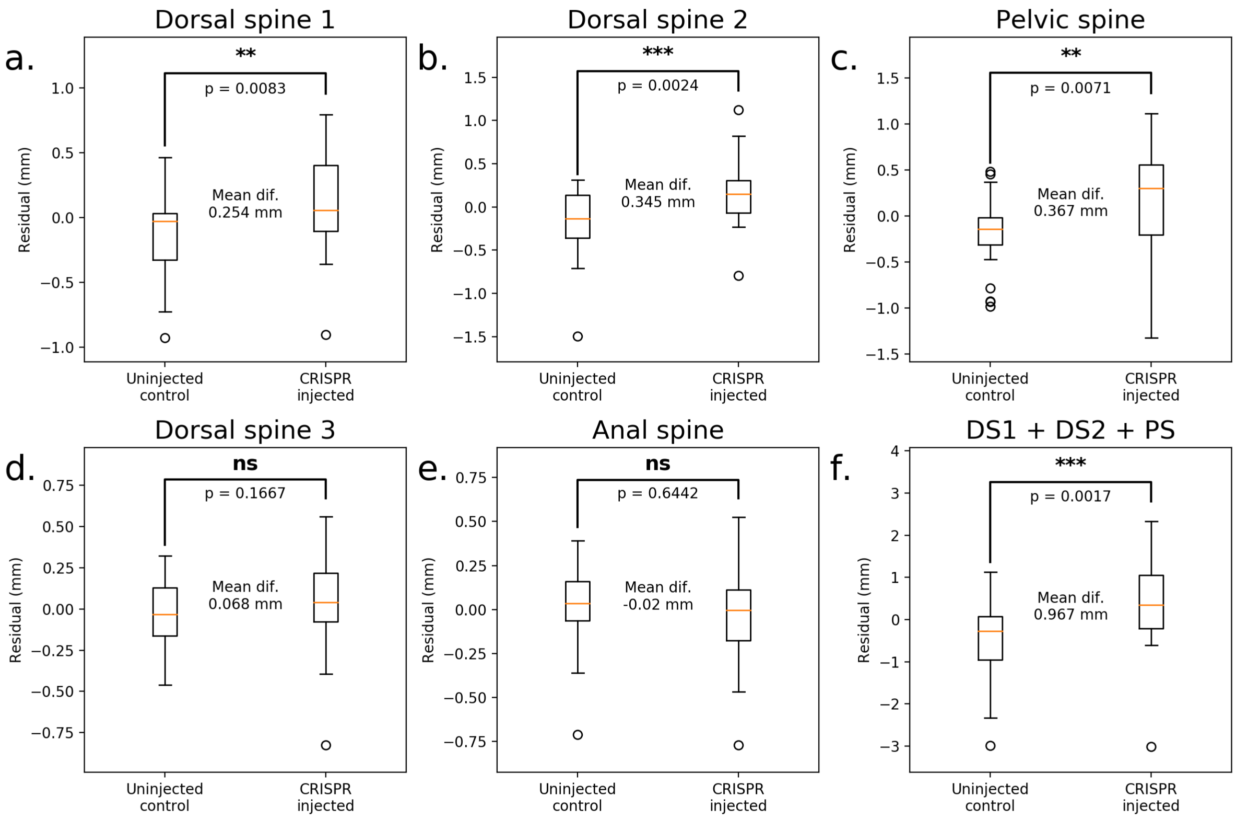

Fig. S7. CRISPR targeting of *Stc2*a results in increased lengths of major spines in mosaic founders. Injected fish have significantly longer first dorsal spines (a), second dorsal spines (b), and pelvic spines (c), but do not show significant differences in third dorsal spines (d) or anal spines (e). Sample sizes: n=23 controls, and n=26 injected fish (Table S12).

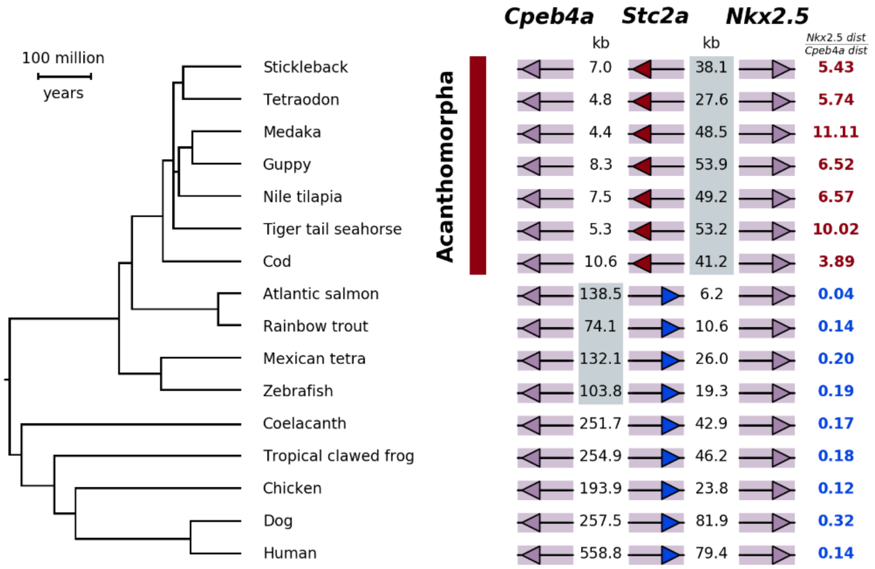

Fig. S8. Orientation and spacing changes around *Stc2a* in acanthomorphs (red). Arrows represent gene orientation for *Stc2a* and its immediate neighbors. Intergenic distances are listed between adjacent loci for each species. The vertical grey bars represent the interval that contains *Maser* orthologous sequences (which are not identifiable outside ray-finned fish). Note that an inversion of *Stc2a* in Acanthomorpha results in a large increase in absolute (middle column) and relative distance (right column) to *Nkx2.5.*

Dataset S1 (separate file). Spine and standard length measurements in fish with previously sequenced genomes (5).

Dataset S2 (separate file): All triallelic regions identified in the Pacific Northwest.

Dataset S3 (separate file): Gene ontology enrichments for genes in triallelic regions and the closest one on each side.

Dataset S4 (separate file): Markers used in *Maser* association mapping analysis.

Dataset S5 (separate file): Genotypes at markers across *Maser* and all spine length residuals after regression against standard length and sex.

Dataset S6 (separate file): Percent Variance Explained (PVE) and Dominance at the two peak markers in *Maser*. Dominance is defined here as the heterozygous phenotype being the same as Long/Long at h=1 and the same as Short/Short at h=0. These values are reported as observed in the wild populations and may differ from those that a traditional QTL cross would yield due to the different allelic ratios. Specifically, the Short/Short genotype is relatively rare in these samples, leading to an underestimation of PVE.

Dataset S7 (separate file): Allele-specific RNA-seq read counts in all fish and all tissues at all five *Stc2a* SNPs analyzed. Short (Bear Paw) x Marine (Rabbit Slough) samples are denoted as "BP" while Long (Mayer Lake) x Marine (Rabbit Slough) samples are denoted as "M". Tissues analyzed are dorsal spines (DS), pelvic spines (PS), pectoral fins (PF), and heart (H).

Dataset S8 (separate file): Gene ontology analysis on genes called as differentially expressed between the two crosses using DESeq2 (potential *trans* downstream genes).

Dataset S9 (separate file): All PCR primer sequences.

Dataset S10 (separate file): Gene ontology analysis on genes showing reciprocal expression changes.

Dataset S11 (separate file): Population group assignments for selected triallelic regions.

Dataset S12 (separate file): Spine measurements of CRISPR-injected and control stickleback.

SI References

1. T. R. Howes, B. R. Summers, D. M. Kingsley, Dorsal spine evolution in threespine sticklebacks via a splicing change in *MSX2A*. *BMC Biol.* **15**, 115 (2017).

2. M. Higuchi, A. Goto, Genetic evidence supporting the existence of two distinct species in the genus *Gasterosteus* around Japan. *Environ. Biol. Fishes* **47**, 1–16 (1996).

3. Y. F. Chan, *et al.*, Adaptive evolution of pelvic reduction in sticklebacks by recurrent deletion of a *Pitx1* enhancer. *Science* **327**, 302–305 (2010).

4. F. C. Jones, *et al.*, The genomic basis of adaptive evolution in threespine sticklebacks. *Nature* **484**, 55–61 (2012).

5. G. A. Roberts Kingman, *et al.*, Predicting future from past: The genomic basis of recurrent and rapid stickleback evolution. *Science Advances* **7**, eabg5285 (2021).

6. P. F. Colosimo, *et al.*, Widespread parallel evolution in sticklebacks by repeated fixation of ectodysplasin alleles. *Science* **307**, 1928–1933 (2005).

7. C. T. Miller, *et al.*, cis-Regulatory changes in Kit ligand expression and parallel evolution of pigmentation in sticklebacks and humans. *Cell* **131**, 1179–1189 (2007).

8. V. B. Indjeian, *et al.*, Evolving new skeletal traits by *cis*-regulatory changes in bone morphogenetic proteins. *Cell* **164**, 45–56 (2016).
